# Phase I/II evaluation of RV1001, a novel PI3Kδ inhibitor, in spontaneous canine lymphoma

**DOI:** 10.1371/journal.pone.0195357

**Published:** 2018-04-24

**Authors:** Heather L. Gardner, Sarah B. Rippy, Misty D. Bear, Kim L. Cronin, Heather Heeb, Holly Burr, Claire M. Cannon, Kumar V. Penmetsa, Srikant Viswanadha, Swaroop Vakkalanka, Cheryl A. London

**Affiliations:** 1 Sackler School of Graduate Biomedical Sciences, Tufts University, Boston, Massachusetts, United States of America; 2 University of Missouri, Columbia, Missouri, United States of America; 3 Kansas State University, Manhattan, Kansas, United States of America; 4 New England Veterinary Oncology Group, Waltham, Massachusetts, United States of America; 5 Blue Pearl Kansas City, Overland Park, Kansas, United States of America; 6 Las Vegas Veterinary Specialty Center, Las Vegas, Nevada, United States of America; 7 Department of Veterinary Clinical Sciences, University of Minnesota, St. Paul, Minnesota, United States of America; 8 Rhizen Pharmaceuticals SA, Switzerland; 9 Incozen Therapeutics Pvt. Ltd., Hyderabad, India; 10 Cummings School of Veterinary Medicine, Tufts University, Grafton, Massachusetts, United States of America; Colorado State University, UNITED STATES

## Abstract

**Background:**

RV1001 is a novel, potent, and selective PI3Kδ inhibitor. The purpose of this study was to evaluate the safety and efficacy of RV1001 in canine Non-Hodgkin lymphoma (NHL).

**Methods and results:**

Inhibition of endogenous pAKT by RV1001 in primary canine NHL cells was determined by Western blotting. A phase I study of RV1001 was performed in 21 dogs with naïve and drug resistant T and B-cell NHL to assess safety, pharmacokinetic profile, and response to therapy. The objective response rate was 62% (complete response (CR) n = 3; partial response (PR) n = 10), and responses were observed in both naïve and chemotherapy-resistant B and T cell NHL. This study provided the recommended starting dose for a phase II, non-pivotal, exploratory, open label multi-centered clinical trial in 35 dogs with naïve and drug resistant T and B-cell NHL, to further define the efficacy and safety profile of RV1001. The objective response rate in the phase II study was 77% (CR n = 1; PR n = 26). Clinical toxicities were primarily hepatobiliary and gastrointestinal, and were responsive to dose modifications and/or temporary drug discontinuation. Hepatotoxicity was the primary dose limiting toxicity.

**Conclusions:**

RV1001 exhibits good oral bioavailability, an acceptable safety profile, and biologic activity with associated inhibition of pAKT in dogs with B and T cell NHL. Data from these studies can be leveraged to help inform the design of future studies involving isoform-selective PI3K inhibitors in humans.

## Introduction

Phosphatidylinositol 3-kinase (PI3K) is an intracellular lipid kinase central to many cell processes through its action as a second messenger, including cell growth and survival, motility, and entry into the cell cycle. Activation of the PI3K pathway is mediated by class I isoform (α, β, γ and δ) signaling through the second messenger PIP3, resulting in activation of downstream targets including AKT and mTOR [1, 2]. While α and β isoforms are ubiquitous in their distribution, expression of δ and γ isoforms are largely restricted to hematopoietic cells [3–5].

Whole genome and whole exome sequencing of human diffuse large B-cell lymphoma and canine T-cell lymphoma have identified recurrently mutated genes related to dysregulation of the PI3K/PTEN signaling axis [6, 7]. In addition, inhibition of PI3Kδ isoforms have been shown to modulate the function of T-regulatory cells, with subsequent upregulation of cytotoxic T-cells [8, 9], supporting the notion that selective targeting of PI3Kδ is a promising approach in the treatment of lymphoma with effects on both the primary tumor cells and the tumor microenvironment. Given the high incidence of dysregulation of PI3K and its downstream effectors in cancer, PI3K is an attractive target for therapeutic intervention.

Several PI3K isoform-selective inhibitors, including CAL-101 and AMG319, have been investigated *in vitro* and in clinical trials in hematologic malignancies [10–13]. Idelalisib (Zydelig; formerly GS-1101, CAL-101), an oral selective PI3Kδ inhibitor, received FDA approval in 2014 for the treatment of relapsed/refractory B-CLL (in combination with rituximab), relapsed follicular B-cell lymphoma, and relapsed small lymphocytic lymphoma. PI3Kδ inhibition has been shown to induce apoptosis, down regulate phosphorylated AKT and modulate tumor microenvironment-mediated chemokine signaling in hematologic malignancies [12–14]. Despite marked clinical successes, idelalisib and other PI3K inhibitors have several limitations including side effects such as hepatotoxicity and gastrointestinal toxicity [15, 16]. RV1001 (Rhizen Pharmaceuticals) is an orally bioavailable potent and selective PI3Kδ inhibitor with strong hinge binding interaction at Val-882. RV1001 inhibits growth of B-cell and T-cell lymphoma cell lines in a δ-isoform selective manner and exhibits anti-cancer activity in murine xenograft tumor models [17].

The ultimate goal of this body of work was to investigate the clinical efficacy of a novel and selective PI3Kδ inhibitor, RV1001, in a spontaneous large animal model of lymphoma to help inform the development of isoform specific PI3K inhibitors in patients with non-Hodgkin lymphoma (NHL). Given the genetic and biologic similarities of NHL in dogs and people, clinical responses to PI3K inhibitor treatment in dogs with NHL are expected to accurately predict responses and toxicities in humans. The activity of RV1001 was first assessed *ex vivo* in canine primary lymphoma cells. Phase I and II clinical trials of the novel PI3Kδ inhibitor RV1001 were subsequently completed in dogs with spontaneous NHL to determine the biologic activity and clinical toxicities associated with RV1001.

## Materials and methods

### Ex vivo treatment of primary tumor samples

Fine needle aspiration of peripheral lymph nodes was performed in dogs with naïve and drug resistant NHL. Aspirates were obtained in the setting of disease progression, prior to administration of steroids or chemotherapeutic agents. Aspirates were placed in sterile PBS. Cells were immediately washed and maintained at 37°C in humidified 5% CO_2_ for 24 hours with vehicle (DMSO) or RV1001 (1 μM) in RPMI 1640 medium (Gibco) containing 20% fetal bovine serum (FBS), 1 mM sodium pyruvate (Gibco), non-essential amino acids (Gibco), 10 mM HEPES buffer (Gibco), 1x Glutamax, and 1x pencillin/streptomycin (Gibco). Cells were collected after 24 hours and stored at -80°C for western blotting.

### Western blotting

Samples were resuspended in fresh lysis buffer consisting of 20 mM Tris-HCl pH 8.0, 137 mM NaCl, 10% glycerol, 1% IPEGAL CA-630, 10 mM ethylenediaminetetraacetic acid (EDTA), 1 mg/mL aprotinin, 1 mg/ml leupeptin, 1 mg/mL pepstatin A, 1 mM phenylmethylsulphonyl fluoride, 1 mM sodium orthovanadate, and 10 mM sodium fluoride (all from Sigma, St. Louis, MO). Samples were vortexed, then rocked for 1 hour at 4°C. Protein lysates were collected, quantified using the Bradford assay, and 80 μg of protein per sample was separated by SDS-PAGE, and transferred to a PVDF membrane. Membranes were incubated overnight at 4°C with a 1:1000 dilution of anti-pAkt (Cat# 9271; Ser473, Cell Signaling Technology, Davers, MA). The membranes were incubated with goat-anti-rabbit horseradish peroxidase (HRP) linked secondary antibody (diluted 1:20,000; Pierce; Cat#: 1858415) at room temperature for 1h, washed, and exposed to substrate (SuperSignal West Dura Extended Duration Substrate, Pierce, Rockford, IL). Blots were stripped, washed, and reprobed for total Akt using a 1:500 dilution on a rotating platform overnight at 4°C (Cat# 610861; BD Transduction Laboratories^™^, San Jose, CA); goat-anti-mouse HRP linked secondary antibody (Cat# 1858413; Pierce) was used at a concentration of 1:20,000 as indicated above. β-actin (Cat# 1616; Santa Cruz Biotechnology, Santa Cruz, CA) was used as a loading control at a 1:1000 dilution as indicated above; Donkey-anti-goat HRP linked secondary antibody (Cat# 2020; Santa Cruz Biotechnology, Santa Cruz, CA) was used at a concentration of 1:20,000 as indicated above. Protein lysates obtained from a canine mast cell tumor line, C2, were used as a positive control for AKT (data not shown).

### RV1001 formulation

RV1001 was supplied in gelatin capsules of 25 mg, 100 mg and 500 mg active ingredient using pharmaceutical grade excipients for the Phase I study. In the Phase II study RV1001 was supplied as 25 mg, 100 mg, 250 mg and 400 mg active ingredient capsules. Capsules were stored at room temperature protected from light.

### Clinical trial design

This was a non-randomized, open label evaluation of RV1001, a PI3K δ isoform selective inhibitor, administered as a single agent to client-owned dogs with spontaneous B-cell and T-cell NHL. In the phase I dose-escalation study, dogs were assessed for tumor response and clinical toxicities every 7 days for the first 28 days, then every 2 weeks thereafter. The initial dose of RV1001 was 10 mg/kg by mouth once daily with escalation planned up to 50 mg/kg in cohorts of 3 until the dose limiting toxicity (DLT) was identified.

After preliminary identification of the activity and safety of RV1001, an open label, non-randomized single-agent, multicenter phase II clinical trial was undertaken to better define the safety and activity of RV1001 at doses below the MTD. RV1001 was administered at a starting dose of 15 mg/kg orally for 5 consecutive days, followed by 2 days off the drug. Dose increases in 5 mg/kg increments up to 25 mg/kg were permitted in patients that did not respond to 15 mg/kg RV1001. A single dose decrease to 10 mg/kg was permitted to maintain the dose schedule if indicated. Dogs were assessed for clinical response and adverse events (AEs) every 7 days for the first 28 days, then every 2 weeks thereafter.

In both the phase I and phase II studies, routine hematologic and biochemical profiles were performed at each study visit. The DLT in both studies was defined as any grade 3 or 4 toxicity, or any chronic toxicity that significantly limited quality of life, as classified by the VCOG-CTCAE criteria v1.1 [18]. The maximum tolerated dose (MTD) was considered to be one dose below that in which the DLT was noted. Clinical toxicities related to disease progression or other unrelated comorbid conditions were not considered drug-related AEs.

### Clinical trial eligibility and ethics statement

The Clinical Research Committee at the College of Veterinary Medicine at The Ohio State University (OSU) and the Institutional Animal Care and Use Committee (IACUC) at OSU approved this phase I study. Animal Clinical Investigation (ACI) Animal Care and Use Committee (ACUC) or equivalent institutional approval was also obtained for the multicenter phase II study at all sites participating in the clinical trial, including OSU, University of Minnesota, New England Veterinary Oncology Group (Waltham, MA), Blue Pearl Kansas City (Overland Park, KS) and Las Vegas Veterinary Specialty Center (Las Vegas, NV). Informed consent was obtained from all owners prior to study entry. Appropriate steps were taken to alleviate any suffering or distress during the course of the clinical trial, including sedation, analgesics and appropriate supportive care as indicated in each dog.

To be considered for enrollment, dogs were required to have a histologic or cytologic diagnosis of B-cell or T-cell NHL that had failed standard therapy, or for which no alternative therapy existed or was declined by the owner. Immunophenotype was determined using flow cytometry on fine needle aspiration samples from an affected lymph node or immunohistochemistry following lymph node biopsy. Additional required eligibility criteria included ≥ 1 year of age at the time of enrollment; adequate organ function as determined by standard laboratory evaluation; discontinuation of any other investigational drug within 1 week of study entry; and no other serious systemic disorder incompatible with the study. For dogs presenting with relapsed disease, prior antineoplastic therapy must have been completed 2 weeks prior to study entry with complete recovery from associated toxicities.

### Tumor response assessment

Response assessments were performed at enrollment and at each scheduled study visit (days 0, 7, 14, 21, 28 and every 14 days thereafter). Responses were characterized according to the VCOG response evaluation criteria for peripheral nodal lymphoma (v1.0) by assessment of peripheral lymph nodes and/or thoracic radiography [19]. An objective response consisted of either a complete or partial response. A complete response (CR) was defined as complete resolution of all target lesions. Partial response (PR) was defined as ≥ 30% reduction in the sum of the longest dimensions of the target lesions, taking as a reference the baseline sum of the longest dimensions. Progressive disease (PD) was defined as a > 20% increase in the sum of the longest dimensions of the target lesions, taking as a reference the smallest sum of the longest dimensions since treatment initiation, or the appearance of at least 1 new non-target lesion. Stable disease (SD) was defined as neither sufficient decrease nor increase in target lesions to be considered an objective response or disease progression, respectively.

### Concomitant medications

Concomitant medications to prevent and/or treat clinical toxicities were used at the discretion of the attending clinician and included antiemetics (metoclopramide, ondansetron, maropitant), antacids (famotidine, randitidine, omeprazole), anti-diarrheals (bismuth subsalicylate, loperamide), analgesics (butorphanol, tramadol, fentanyl), antihistamines (diphenhydramine) and liver protectants (S-adenosylmethionine and silybin). Prednisone use was permitted at enrollment in the phase I and phase II study if dogs had been receiving prednisone for at least 2 weeks prior to study entry and experienced disease progression while on prednisone. However, in the phase II study, tapering and discontinuation of prednisone by day 28 of the study was attempted in all dogs receiving prednisone at the time of study enrollment.

### RV1001 pharmacokinetics

Plasma samples were taken to assess blood levels of RV1001 on day 0 prior to dosing and at 0, 2, 4, 6 and 8 hours post-dosing on Day 0 of the phase I study. Trough plasma samples were also obtained on days 7, 14, 21 and 28 prior to RV1001 administration. In addition, trough plasma samples were obtained from 10 dogs in the phase II study at day 0 prior to RV1001 administration and then after the 2-day RV1001 washout on days 7, 28, 56 and 84. Approximately 2 ml of blood was placed in an EDTA tube. Blood samples were centrifuged at room temperature for 10 minutes, within 20 minutes of collection. Plasma was transferred to cryovials and stored at -80°C until analysis. Plasma samples were analyzed for RV1001 using a validated LC/MS/MS method over a concentration range of 20–14700 ng/mL. Briefly, extraction of RV1001 from dog plasma was carried out by protein precipitation with acetonitrile. Chromatographic separation of analytes was performed on a Phenomenex Kinetex 100A (100 mm × 4.6 mm, 2.6 μm) column under isocratic conditions with acetonitrile: 0.1% formic acid buffer as the mobile phase at a flow rate of 0.55 mL/min. Precursor ion and product ion transition for analyte was monitored on a triple quadrupole mass spectrometer. Pharmacokinetic parameters i.e. C_max_, AUC_0-t_, Tmax, and t_½_ were estimated by WinNonlin (Phoenix 6.1 software) using a non-compartmental analysis method.

### Evaluation of primary tumor cells for modulation of phospho-AKT

Fine needle aspiration samples were obtained from peripheral lymph nodes to assess target modulation *in vivo* at pre-dose and 2 hours after RV1001 administration on Day 0 and before treatment on Days 7 and 21 of the Phase I study. Samples were placed directly into cryovials and flash frozen in liquid nitrogen. Cryovials were stored at -80°C until analysis. Frozen samples were pulverized into a powder while in liquid nitrogen. Tissue powder was thawed briefly on ice and resuspended in fresh lysis buffer. Western blot for pAKT, total AKT and β-actin was performed as above.

### Statistical analysis

The number of dogs enrolled in the phase II study was estimated assuming 80% power and a 95% confidence, with 17 dogs in each group necessary to demonstrate a 30% difference in the mean sum of the longest diameter of target lesion measurements. Patient characteristics were summarized by treatment groups. Time to progression (TTP) was defined as the time between study entry and PD. Dogs were censored from the progression analysis if they were lost to follow-up, or did not have documented disease progression at the time of euthanasia. Duration of response was defined as the time period between the first of two evaluations demonstrating an objective response (CR+PR) and/or stable disease (clinical benefit; SD+PR+CR) until the date of disease progression or death. Only dogs with SD noted for at least 2 sequential visits were considered to have clinical benefit (CB). Data were analyzed using SAS version 9.2 (Cary, NC) and summarized. Descriptive summary statistics including best overall response rate (ORR, best response of PR or better), disease control rate (best response of SD or better), and duration of response (DOR) were considered. Rates ± 95% confidence intervals were determined. Log rank mantel cox regression analysis was used to test for differences in TTP between categorical groups. P-value < 0.05 was considered significant. Progression analyses were performed in GraphPad Prism 7 (GraphPad Software, Inc. La Jolla, CA).

## Results

### Ex vivo target modulation of RV1001 in canine primary lymphoma cells

**Fig 1:**
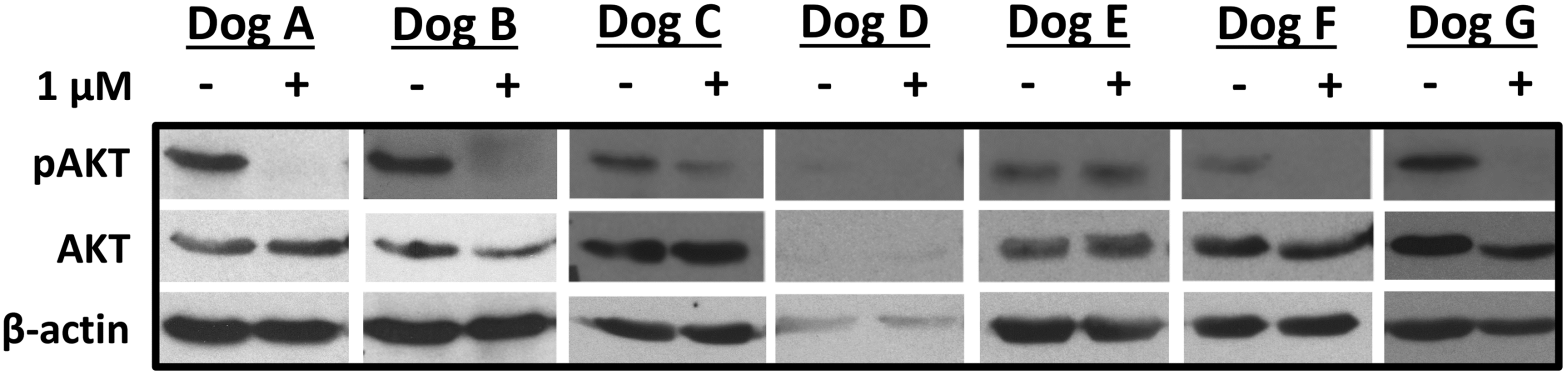
Biologic activity of RV1001 against canine primary NHL cells treated *ex vivo*. Inhibition of pAKT in primary canine NHL cells treated *ex vivo* with RV1001 is demonstrated by western blotting.

The effects of RV1001 were evaluated *ex vivo* on canine primary lymphoma cells obtained from dogs with naïve and drug resistant NHL. Basal expression of AKT and pAKT were detected in 6 of 7 dogs evaluated. In one dog (Dog D), decreased β-actin precluded complete evaluation of AKT expression. Inhibition of pAKT was noted in 5 of 7 samples treated *ex vivo* with RV1001 for 24 hours (Fig 1).

### Phase I: MTD and determination of recommended dose for phase II study

A total of 21 dogs (n = 13 dose escalation; n = 8 dose expansion) were enrolled into this phase I clinical trial. Overall patient demographics for both the Phase-1 and Phase-2 trials are detailed in Table 1. The median age for all 21 dogs enrolled in the Phase-1 trial was 8 years, and the median weight was 27.8 kg. The majority of dogs had B-cell lymphoma (n = 8 naïve; n = 7 relapsed). Twelve dogs were receiving prednisone at the time of enrollment. In these cases, disease progression occurred during prednisone administration and therefore prednisone was not discontinued. An additional 2 dogs were placed on prednisone after enrollment on day 1. Prednisone was initiated in one dog due to anorexia and diarrhea secondary to lymphoma on day 1. In this dog, prednisone was discontinued by day 14 of the study, and this dog obtained a CR after discontinuation of prednisone. In another dog, prednisone was initiated on day 21 after development of PD while RV1001 treatment was temporarily discontinued due to an adverse event.

**Table 1:** Demographics.

Characteristics	Phase I Dose Escalation	Phase I Dose Expansion	Phase II	All Dogs
Number of Dogs	9	12	35	56
**Median Age (years)**	9	7.5	7	7.5
Range	3–12	3–12	4–13	3–13
**Median Weight (kg)**	28.8	27	25.6	27.7
Range	5.45–34.2	9.4–50	6.2–53	5.45–53
**Gender**				
Male Intact	2	1	2	5
Male Castrated	6	4	16	26
Female Intact	0	2	1	3
Female Spayed	1	5	16	22
**Breed**				
Mixed Breed	2	2	8	12
Labrador Retriever	0	3	1	4
Boxer	2	1	5	8
Golden Retriever	1	1	7	9
Other Purebred Dogs	4	5	14	23
**Immunophenotype**				
B-cell (Naïve)	4	4	15	23
B-cell (Relapsed)	2	5	7	14
T-cell (Naïve)	1	1	5	7
T-cell (Relapsed)	2	2	8	12
**Prior Chemotherapy**				
Yes	4	7	15	26
No	5	5	20	30
**Prior Prednisone**				
Yes	4	8	15	27
No	5	4	20	29

#### Treatment groups

Nine dogs were initially entered into the Phase-1 clinical trial at escalating doses of RV1001 given once daily. RV1001 dosing groups were as follows: 10 mg/kg PO q24h, 15 mg/kg PO q24h and 25 mg/kg PO q24h. An additional 3 cohorts of dogs (n = 12 dogs total; n = 4 dogs per cohort) were subsequently enrolled and administered RV1001 at an altered dosing regimen with the goal of decreasing drug accumulation. RV1001 dosing for the expansion cohorts was 15 mg/kg PO q24h Monday through Friday (M-F), 25 mg/kg PO q24h M-F (chemotherapy naïve dogs only) and 25 mg/kg PO q24h M-F (relapsed dogs only) (Tables 2 and 3). This regimen was instituted due to hepatotoxicity that developed 2–3 weeks after initiation of therapy in conjunction with high trough plasma levels of RV1001 (Table 3).

**Table 2:** Adverse events.

Dose (mg/kg)	No. of Dogs	Vomiting				Diarrhea				Anorexia				Weight Loss			
AE Grade		1	2	3	4	1	2	3	4	1	2	3	4	1	2	3	4
10	3		1*			1	1			2	1						
15	3	2								1	2			1			
25	3	2				2	2			2	3			2			
15 M-F	4		1							1							
25 M-F (Relapsed)	4	1				2											
25 M-F (Naïve)	4	1				1	1			2	1			1	1		

* Number of observed events

**Table 3:** Biochemical adverse events.

Dose (mg/kg)	No. of Dogs	Elevated ALT				Elevated AST				Elevated ALP				Elevated Bilirubin			
AE Grade		1	2	3	4	1	2	3	4	1	2	3	4	1	2	3	4
10	3				2*		1	1		1		1		1	1		
15	3				2	2		1		1		1	1	1			1
25	3		1	1				2		1		2					
15 M-F	4	1	1	1		1		1		1							
25 M-F (Relapsed)	4				1			1				1					
25 M-F (Naïve)	4		1	2	2	1		4		3		1					1

* Number of observed events

#### Pharmacokinetic analysis

**Fig 2:**
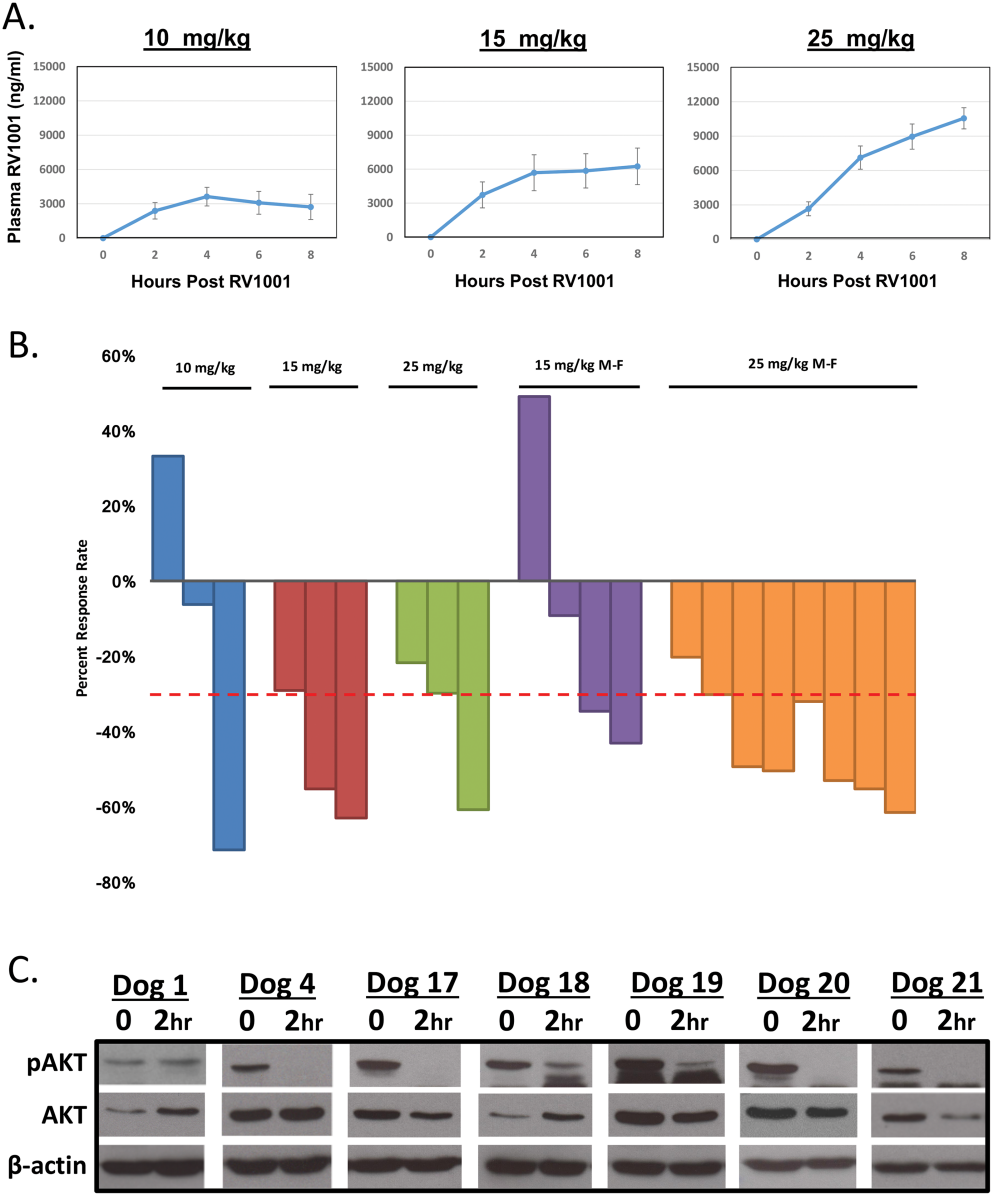
Response to RV1001 administration in dogs with NHL. **(A)** Blood samples were collected to assess plasma concentrations of RV1001 over an 8 hour period after RV1001 administration. **(B)** Dogs were evaluated once weekly for response assessments. Objective responses were noted in all dosing cohorts. **(C)** Lymph node samples were collected from dogs before and after RV1001 administration. Inhibition of pAKT was observed within 2 hours of drug administration. Dosing groups: Dog 1 (10 mg/kg); Dog 4 (15 mg/kg); Dogs 18, 19, 20, 21 (25 mg/kg M-F).

Pharmacokinetic analysis of RV1001 was performed in all dogs on the first day of drug administration. Blood samples were collected to assess plasma concentrations of RV1001 over an 8-hour period after RV1001 administration. In addition, blood samples were collected prior to drug administration on days 7, 14, 21 and 28 (data not shown). Pharmacokinetic results are shown in Fig 2A. A dose proportional increase in drug exposure was observed in dogs after one dose of RV1001, however some variability was noted in both Cmax and Tmax. Half-life could not be determined since RV1001 concentrations were persistently high in plasma at the 8-hour time point.

#### Adverse events and maximum tolerated dose

Concomitant medications used to treat adverse events associated with RV1001 administration and/or disease-related adverse events included the following: maropitant, ondansetron, intravenous fluids (lactated ringers, plasmalyte), omeprazole, famotidine, sucralfate, metronidazole, metoclopramide, fortiflora, pantoprazole, S-adenosylmethionine/silybin.

Adverse events (AEs) during treatment consisted primarily of elevations in liver transaminases and mild gastrointestinal toxicity (Tables 2 and 3). In 5 dogs, temporary RV1001 discontinuation (range: 2–4 days) was required for mild gastrointestinal toxicity consisting of grade 1 and 2 anorexia, vomiting and diarrhea. Gastrointestinal AEs were responsive to medical management and temporary RV1001 discontinuation, and therapy was resumed without necessitating changes in the therapeutic regimen. Three dogs experienced hematologic AEs. Grade 1 anemia was noted in one dog secondary to gastrointestinal toxicity. Grade 4 thrombocytopenia and grade 1 neutropenia were observed in two dogs in the setting of progressive disease while receiving RV1001.

During the dose escalation phase of the clinical trial, the dose limiting toxicities (DLT) were grade 3 and 4 transaminase elevations. Hepatotoxicity, as evidenced by elevations in liver transaminases, was noted in all dosing groups, typically after 2–3 weeks of drug administration, and was associated with high trough blood levels of RV1001 (20–30 μM). Due to the timeframe in which hepatotoxicity developed, dose escalation in the study had commenced. Temporary RV1001 discontinuation (range: 1–3 weeks) was required in 5 dogs during the dose escalation phase of the study due to elevations in liver transaminases. A modified dosing schedule was instituted in which RV1001, administered once daily Monday through Friday (M-F), was investigated in an attempt to abrogate the observed hepatotoxicity based on interim PK analysis demonstrating drug accumulation. This regimen was well tolerated in most dogs with hepatotoxicity occurring in 2 dogs treated M-F. Hepatotoxicity was reversible upon drug discontinuation in all but one dog. Therefore, the maximum tolerated dose (MTD) was determined to be 15 mg/kg M-F.

### Phase II: Safety and efficacy of RV1001

A total of 35 dogs (n = 24 enrolled at 15 mg/kg PO q24h for 5 consecutive days; n = 11 enrolled at 10 mg/kg PO q24h for 4 consecutive days) were enrolled into the phase II clinical trial. Patient demographics are detailed in Table 1. The median age for all 35 dogs was 7 years, and the median weight was 25.6 kg. Twenty-two dogs had B-cell lymphoma (n = 15 naïve; n = 7 relapsed) and 13 dogs had T-cell lymphoma (n = 5 naïve; n = 8 relapsed). While the initial intent of this clinical trial was to follow the dosing regimen established in the phase 1 study (15 mg/kg 5 days on/2 days off) the persistence of hepatotoxicity resulted in institution of a 4 days on/3 days off regimen intended to provide a more sustained drug washout and thus eliminate issues with drug accumulation. However, ten dogs enrolled at 15 mg/kg M-F required a dose reduction to 10 mg/kg due to the development of adverse events, primarily consisting of hepatotoxicity. Because hepatotoxicity was still noted in a proportion of dogs, a second cohort of dogs (n = 11) were enrolled at 10 mg/kg M-Th. No dogs enrolled at 10 mg/kg M-Th required a dose reduction. This did appear to mitigate the toxicity as only 1/5 dogs with grade 3 or 4 enzyme or bilirubin elevations at this dosage was subsequently found to be directly drug related due to resolution following discontinuation of RV1001.

#### Pharmacokinetic analysis

Pharmacokinetic analysis was performed in a subset of dogs (n = 5 each treated at 15 mg/kg M-F and 10 mg/kg M-Th) to determine trough plasma levels of RV1001 after a dosing cycle. In all dogs evaluated, the trough RV1001 plasma levels were below the limit of detection, indicating that the 3-day drug washout was sufficient to prevent drug accumulation.

#### Adverse events

All AEs noted during the Phase II study are reported in S1 Table. These consisted primarily of elevations in liver transaminases and low grade gastrointestinal toxicity, similar to the clinical toxicities noted in the phase I dose escalation study. Ten dogs receiving RV1001 at 15 mg/kg M-F required a dose reduction to 10 mg/kg M-F due to the development of elevations in liver transaminases. Drug-related hematologic AEs were rare, with grade 3 (n = 1) and 4 (n = 1) thrombocytopenia and grade 3 neutropenia (n = 1) observed. Twenty-five drug-related gastrointestinal AEs were reported; however, all were grade 1 and 2. Twenty-two of the reported 25 grade 3 and 4 AEs were elevations in liver transaminases or bilirubin (n = 11 ALT, n = 7 AST, n = 2 ALP, n = 2 bilirubin).

Of the dogs enrolled at 15 mg/kg, 9/24 dogs had grade 3 or 4 ALT elevations; of those, 4 were concordant with disease progression and one was confirmed at necropsy to have diffuse hepatic lymphoma and no evidence of drug toxicity. Of the 4 dogs that were followed to determine resolution of enzyme or bilirubin elevation, 3 resolved with drug discontinuation and therefore were definitively linked to treatment. At 10 mg/kg dosing, 5/11 dogs had grade 3 or 4 ALT elevations; all were concordant with disease progression, with one dog confirmed to have hepatic lymphoma. Of the 2 dogs that were followed to determine resolution of enzyme or bilirubin elevation, one resolved with drug discontinuation and was therefore definitively linked to treatment. Therefore, the lower dosage of drug is associated with a lower rate of liver enzyme or bilirubin elevations during treatment.

Three dogs experienced severe adverse events (SAE) during the study. One dog developed rapid progression of lymphoma within the first week of the study and died at home. Two additional dogs developed grade 3 and 4 hepatotoxicity and were subsequently euthanized via standard intravenous injection. Both dogs were subsequently determined to have hepatic lymphoma, with no histopathologic evidence of drug toxicity in the hepatic parenchyma on post-mortem histopathologic examination.

#### Response to therapy

The median time to progression (TTP) in the phase I study was 21 days (range 7–98 days). The objective response rate (n = 3 CR; n = 10 PR) was 62%, with a median duration on study of 28 days (range 6–125 days) (Fig 2B). Notably, all dogs with T-cell lymphoma (n = 2 naïve; n = 4 chemotherapy-resistant) derived clinical benefit (2 CR, 3 PR, 1 SD) from RV1001 therapy.

Based on the pharmacologic data, adverse event profile, and biologic activity observed during the initial dose escalation, an alternate dosing scheme was investigated to abrogate the observed DLT (elevated liver transaminases), while still providing adequate drug exposure and clinical benefit. Response rates were similar when RV1001 was administered M-F compared to once daily, with 83% and 89% of dogs experiencing clinical benefit, respectively. Corresponding Cmax (μM) and AUC (μM*hr) for M-F dosing regimen at 25 mg/kg were 11.34 μg/ml and 50.17 μM*hr, respectively.

Tumor samples were obtained via fine needle aspiration on day 0 prior to RV1001 administration, and 2 hours post-administration on day 0. Western blotting was performed for pAKT/AKT and β-actin (Fig 2C). Consistent with the *ex vivo* treatment of lymphoma cells with RV1001, downstream target modulation was observed at 2 hours post-RV1001 administration in 6/7 dogs with spontaneous NHL. Notably, target modulation correlated with clinical responses, as the one dog with documented PD did not demonstrate target modulation as determined by western blotting. The remaining 6 dogs demonstrating target modulation experienced clinical benefit (n = 1) or an objective response (PR = 3; CR = 2).

**Fig 3:**
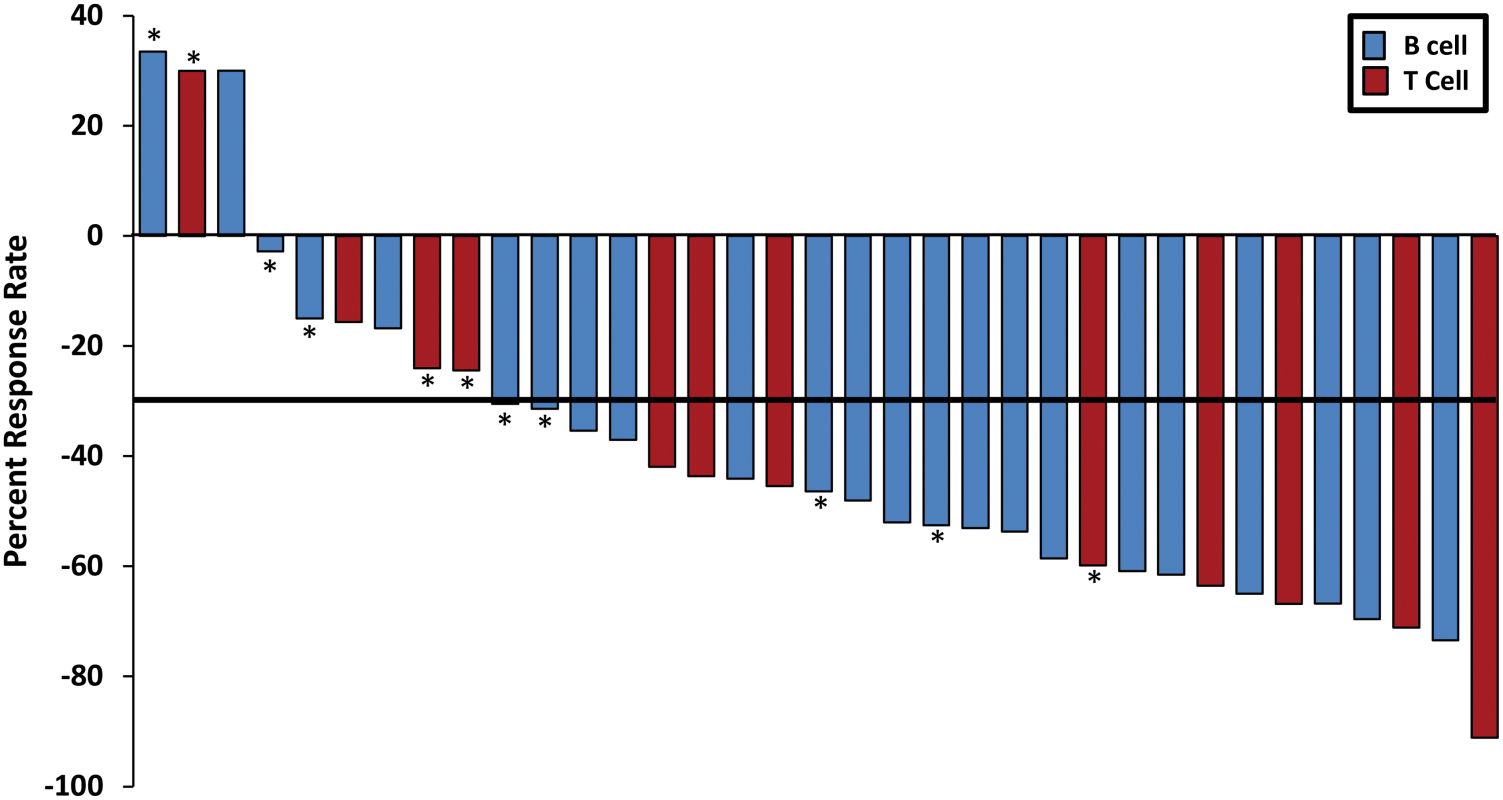
Objective response rates to RV1001 in dogs with NHL. Waterfall plot showing the best ORR for dogs enrolled in the phase II study. Solid horizontal black line indicates a reduction in size of the target lesions of at least 30%, consistent with a partial response. * = dogs enrolled in the clinical trial at 10 mg/kg M-Th RV1001.

While hepatotoxicity was largely averted in the phase I clinical trial at doses not exceeding 25 mg/kg, prolonged hepatic exposure to RV1001 at high micromolar concentrations was associated with hepatic damage. Since response to therapy was noted at all dose levels in the phase I study, and doses of 15 mg/kg achieve peak plasma concentrations averaging 6 μg/ml (range 4–8 μg/ml) (Fig 2A), a starting dose of 15 mg/kg given M-F with a 2-day washout period was chosen for the phase II clinical trial. As discussed above, this was altered to a 4 day on/3 day off (M-Th) regimen due to observed hepatotoxicity. Despite this, some dogs still required a dose reduction to 10 mg/kg on the M-Th regimen due to continued hepatotoxicity. The ORR in the phase II study was 77% (n = 1 CR; n = 26 PR) (Fig 3). Objective responses were noted in both naïve and relapsed NHL, with 86.7% of naïve B-cell NHL, 57.1% of relapsed B-cell NHL, 100% of naïve T-cell NHL and 62.5% of relapsed T-cell NHL obtaining an objective response to RV1001 administration. The overall median TTP in the phase II study was 25.5 days. There was not a significant difference in TTP based on RV1001 dose intensity (p = 0.723), use of RV1001 in naïve or relapsed NHL (p = 0.444) or immunophenotype (p = 0.841) (Table 4).

**Table 4:** Median time to progression.

	10 mg/kg	15 mg/kg	All Dogs
B-cell (Naive)	27^a^ (6–42)	34.5 (14–56)	31 (6–56)
B-cell (Relapsed)	28 (14–42)	24.5 (4–40)	24.5 (4–42)
T-cell (Naive)	ND^b^	24.5 (13–36)	28 (13–36)
T-cell (Relapsed)	21 (1–79)	23 (14–42)	22 (1–79)

^a^ Data reported in days.

^b^ ND: Could not be determined due to only one sample point with a time to progression of 35 days

## Discussion

The PI3K/AKT/mTOR pathway is known to be important in NHL, and AKT is considered a main downstream oncogenic effector in this pathway. AKT phosphorylation is common (>50%) and there is evidence that AKT phosphorylation status is prognostic in human DLBCL [20]. Activation of the pathway occurs through multiple genomic aberrations, such as loss of PTEN, and aberrant CD40L expression on immune cells within the tumor microenvironment [21–23]. Expression of the p110δ isoform is restricted to leukocytes, and is crucial for B-cell development and function [3–5]. Canine and human NHL are genetically and biologically similar. For example, array comparative genomic hybridization (aCGH) has been used to demonstrate shared copy number aberrations in known oncogenic drivers between human and canine diffuse large B-cell lymphoma (DLBCL), and gene expression profiling (GEP) has been used to separate canine lymphoma into categories reminiscent of ABC and GCB molecular subtypes [24, 25]. The similarities between canine and human NHL justify the use of PI3K inhibitors in canine lymphoma to inform subsequent clinical studies in human patients.

Early studies utilizing pan-PI3K inhibitors demonstrated significant off-target effects, limiting their clinical development [26]. However, isoform selective PI3K inhibitors such as idelalisib have demonstrated clinical activity with an acceptable toxicity profile [15]. Notably, activating PI3K mutations *(PIK3CA)* are rare in hematopoietic malignancies in people, accounting for <10% of DLBCL in people [27, 28]. Clinical responses to PI3K inhibition in the absence of driver mutations may be due to PI3K signaling downstream of the B-cell receptor (BCR) or through effects on the tumor microenvironment [8, 29]. Through its close association with the immune system, p110δ modulates the tumor microenvironment by promoting chemokine secretion in malignant cells, facilitating homing and retention of immune cells, such as T-regulatory cells, in the tumor microenvironment [14, 30, 31]. The role of p110δ and γ isoforms in regulating tumor progression, proliferation and apoptosis, in addition to their ability to modulate the tumor microenvironment support interrogation of isoform-selective inhibitors in hematopoietic malignancies. Therefore, the overriding goal of this study was to evaluate the safety, clinical toxicities and pharmacokinetics/pharmacodynamics of a novel PI3Kδ inhibitor, RV1001, in spontaneous canine NHL. Although RV1001 was not evaluated for specific effects in the tumor microenvironment, it is possible that some of the observed clinical effects were due to both tumor cell and microenvironmental effects.

Objective responses were observed in all dose escalation groups. Importantly, objective responses were observed in T-cell and B-cell NHL, as well as naïve and drug-resistant NHL. Single agent overall response rates for relapsed canine NHL range from 27–41% [32–35]. The overall objective response rate in this study was 62%, with 6/11 (55%) relapsed dogs experiencing an objective response, suggesting good single agent activity over traditional chemotherapy agents in the setting of multidrug resistance. Multicentric T-cell lymphoma (LSA) is associated with reduced duration of response to treatment compared to B-cell LSA [36–38]. Importantly, in the phase I study population, the median time to progression (TTP) was longer in dogs with T-cell NHL (49 days) compared to B-cell NHL (14 days), however the difference was not significant (p = 0.067). In addition, the duration of response was not significantly different between B-cell and T-cell NHL in the phase II study (p = 0.841). One preclinical study implicated p110δ in PTEN-deficient T-cell ALL, and utilized a murine model to demonstrate prolonged survival with dual inhibition of γ and δ isoforms [39]. In addition, genetic deletion of both δ and γ isoforms in a murine model resulted in T-cell depletion and diminished function [40]. In summary, while RV1001 predominantly targets the p110δ isoform, activity of other isoforms has been demonstrated as well [17], and some of the biologic activity of RV1001 in T-cell NHL may be due to inhibition of multiple isoforms at the plasma concentrations attained.

Marked objective responses to RV1001 were noted within the first 1–2 weeks of therapy in many dogs. The overall median TTP was 21 days in the phase I study and 25.5 days in the phase II study; however, a subset of dogs experienced prolonged objective responses with ongoing RV1001 administration. Development of resistance early in the course of kinase inhibitor therapy is common. Multiple mechanisms of resistance to PI3K inhibitors have been proposed, including feedback loops and activation of parallel and/or downstream signaling pathways that may result in the development of resistance to PI3K inhibitors. For example, inhibition of the PI3K/AKT pathway led to upregulation of the RAS/MEK/ERK pathway in HER2 overexpressing breast cancer cells in one study [41]. Other pathways implicated in the development of resistance to PI3K inhibitors include MYC and NOTCH [42, 43]. Rational use of combination therapies will likely be necessary to circumvent resistance, and are currently being explored in both humans and dogs to help mitigate the development of drug resistance.

Hepatotoxicity is a reported adverse event associated with PI3K inhibitors [15]. The dose limiting toxicity (DLT) of RV1001 administration was grade 3–4 transaminase elevations (Table 2). Dogs were dosed with RV1001 once daily during the phase 1 study. Hepatotoxicity was related to high (20–30 μM) trough plasma concentrations, consistent with drug accumulation. The dosing regimen was therefore modified to a Monday through Friday (M-F) dosing schedule. This protocol modification was well tolerated and helped abrogate the development of clinically relevant hepatotoxicity without compromising the biologic activity. Based on this data, an expansion study consisting of dogs with naïve and drug resistant LSA (n = 4 each) was undertaken using the modified dosing schedule (M-F). Biologic activity and median TTP were similar in both relapsed and naïve LSA, and objective responses were noted at doses below the MTD. Despite the apparent tolerability of the M-F dosing schedule in the first study, this was not as well tolerated in the phase 2 study, resulting in a change to a Monday-Thursday schedule. This 4 day on/3 day off regimen when administered at 10 mg/kg was well tolerated and resulted in complete drug washout prior to the next dosing cycle (data not shown). Importantly, the dose and regimen change did not compromise the observed responses to therapy. Therefore, while hepatotoxicity is a DLT of RV1001, it is considered manageable with temporary drug discontinuation and/or modification of the dosing regimen, without compromising biologic activity.

One limitation of this study is the concurrent administration of prednisone. This was permitted in dogs receiving prednisone at the time of enrollment that had experienced PD, and every attempt was made to discontinue prednisone administration when possible. In both the dose escalation and dose expansion cohorts, the proportion of dogs that received prednisone was similar. In the phase I study, 1 dog initiated prednisone therapy at day 21 and another received a tapering dose of prednisone (0.5 mg/kg q24h) between day 1 and 14 of the study, due to an AE associated with RV1001. In the phase II study, 8 dogs who were receiving prednisone at the initiation of the study in the face of progressive disease were permitted to continue prednisone after enrollment. Utilization of prednisone primarily in the setting of disease progression and discontinuation of prednisone during the course of the clinical trial reinforces the idea that the observed biologic activity was due to RV1001. However, as prednisone can result in elevations in liver transaminases, it could interfere with assessment of drug-related hepatoxicity.

In conclusion, we have demonstrated that RV1001 administration is associated with objective responses in canine NHL, supporting the notion that PI3Kδ inhibition is a relevant therapeutic target in lymphoid malignancies. Similar response rates were observed in B-cell and T-cell NHL, with several exceptional responders noted, underscoring the importance of rational study design which considers the genetic and molecular context of patient responses, as well as relevant biomarkers implicated in the pathogenesis of hematologic malignancies to identify patients most likely to benefit from PI3K inhibitor therapy.

## Supporting information

S1 Table Adverse events during the phase II clinical trial. All adverse events reported during the phase II study are summarized, based on dose group. n = subject count; E = event count.
(DOCX)
