# Supplementary material for: Phase I/II evaluation of RV1001, a novel PI3Kδ inhibitor, in spontaneous canine lymphoma
Source: PLoS One. 2018 Apr 24;13(4):e0195357. doi: 10.1371/journal.pone.0195357 (PMC5915681; doi:10.1371/journal.pone.0195357)
Supplement: S1 Table — All adverse events reported during the phase II study are summarized, based on dose group. n = subject count; E = event count. (DOCX) [file pone.0195357.s001.docx]

| **Standard Term** | **10 mg/kg** | **15 mg/kg** | **Total** |
| --- | --- | --- | --- |
|  | **(n=11)** | **(n=24)** | **(n=35)** |
|  | **n (%), E** | **n (%), E** | **n (%), E** |
| Any AE | 11 (100.0%), 167 | 24 (100.0%), 442 | 35 (100.0%), 609 |
|  |  |  |  |
| Anorexia | 8 (72.7%), 13 | 21 (87.5%), 40 | 29 (82.9%), 53 |
| Alkaline phosphatase high | 5 (45.5%), 5 | 17 (70.8%), 17 | 22 (62.9%), 22 |
| Vomiting | 7 (63.6%), 17 | 15 (62.5%), 20 | 22 (62.9%), 37 |
| ALT, high | 5 (45.5%), 5 | 14 (58.3%), 17 | 19 (54.3%), 22 |
| AST, high | 6 (54.5%), 6 | 10 (41.7%), 12 | 16 (45.7%), 18 |
| Lymphocytes, low | 5 (45.5%), 7 | 11 (45.8%), 14 | 16 (45.7%), 21 |
| Neutrophils, high | 3 (27.3%), 3 | 13 (54.2%), 16 | 16 (45.7%), 19 |
| Diarrhea | 4 (36.4%), 10 | 9 (37.5%), 22 | 13 (37.1%), 32 |
| Lethargy/fatigue | 5 (45.5%), 5 | 8 (33.3%), 18 | 13 (37.1%), 23 |
| Monocytes, high | 4 (36.4%), 4 | 9 (37.5%), 12 | 13 (37.1%), 16 |
| Weight loss | 2 (18.2%), 2 | 11 (45.8%), 11 | 13 (37.1%), 13 |
| Platelets, low | 1 (9.1%), 1 | 11 (45.8%), 12 | 12 (34.3%), 13 |
| Anemia | 5 (45.5%), 5 | 5 (20.8%), 5 | 10 (28.6%), 10 |
| Cholesterol, high | 2 (18.2%), 2 | 8 (33.3%), 8 | 10 (28.6%), 10 |
| Leukocytes, high | 3 (27.3%), 3 | 7 (29.2%), 9 | 10 (28.6%), 12 |
| Hemoglobin, low | 4 (36.4%), 4 | 5 (20.8%), 5 | 9 (25.7%), 9 |
| Magnesium, low | 0 | 8 (33.3%), 10 | 8 (22.9%), 10 |
| Increased reticulocytes | 4 (36.4%), 4 | 3 (12.5%), 3 | 7 (20.0%), 7 |
| Nucleated RBC | 2 (18.2%), 3 | 5 (20.8%), 7 | 7 (20.0%), 10 |
| Alteration in digestive enzymes | 0 | 6 (25.0%), 8 | 6 (17.1%), 8 |
| Calcium, low | 2 (18.2%), 2 | 4 (16.7%), 4 | 6 (17.1%), 6 |
| Electrolyte balance alteration | 4 (36.4%), 4 | 2 (8.3%), 2 | 6 (17.1%), 6 |
| Fever | 1 (9.1%), 2 | 5 (20.8%), 9 | 6 (17.1%), 11 |
| Lymphocytes, high | 4 (36.4%), 5 | 2 (8.3%), 2 | 6 (17.1%), 7 |
| Total Protein, low | 1 (9.1%), 1 | 5 (20.8%), 5 | 6 (17.1%), 6 |
| Bilirubinemia | 0 | 6 (25.0%), 6 | 6 (17.1%), 6 |
| GGT, high | 2 (18.2%), 2 | 3 (12.5%), 3 | 5 (14.3%), 5 |
| Leukocytes, low | 0 | 5 (20.8%), 5 | 5 (14.3%), 5 |
| Platelets, high | 1 (9.1%), 1 | 4 (16.7%), 5 | 5 (14.3%), 6 |
| Amylase, high | 1 (9.1%), 1 | 3 (12.5%), 3 | 4 (11.4%), 4 |
| Anion GAP, high | 0 | 4 (16.7%), 5 | 4 (11.4%), 5 |
| Band neutrophils | 3 (27.3%), 3 | 1 (4.2%), 1 | 4 (11.4%), 4 |
| BUN, high | 1 (9.1%), 2 | 3 (12.5%), 4 | 4 (11.4%), 6 |
| Decreased CPK | 0 | 4 (16.7%), 4 | 4 (11.4%), 4 |
| Hematocrit, low | 2 (18.2%), 2 | 2 (8.3%), 2 | 4 (11.4%), 4 |
| MCHC, high | 3 (27.3%), 5 | 1 (4.2%), 1 | 4 (11.4%), 6 |
| Albumin, low | 0 | 3 (12.5%), 3 | 3 (8.6%), 3 |
| Chloride, low | 1 (9.1%), 1 | 2 (8.3%), 2 | 3 (8.6%), 3 |
| Constipation | 1 (9.1%), 1 | 2 (8.3%), 2 | 3 (8.6%), 3 |
| Cough | 1 (9.1%), 1 | 2 (8.3%), 2 | 3 (8.6%), 3 |
| Eosinophils, high | 0 | 3 (12.5%), 3 | 3 (8.6%), 3 |
| Eosinophils, low | 2 (18.2%), 2 | 1 (4.2%), 1 | 3 (8.6%), 3 |
| Globulin, low | 0 | 3 (12.5%), 6 | 3 (8.6%), 6 |
| Glucose, high | 2 (18.2%), 2 | 1 (4.2%), 1 | 3 (8.6%), 3 |
| Hypoglycemia | 0 | 3 (12.5%), 3 | 3 (8.6%), 3 |
| Increased BUN/Creatinine Ratio | 0 | 3 (12.5%), 6 | 3 (8.6%), 6 |
| Increased specific gravity of urine | 0 | 3 (12.5%), 3 | 3 (8.6%), 3 |
| Phosphorous, low | 2 (18.2%), 3 | 1 (4.2%), 1 | 3 (8.6%), 4 |
| Potassium, low | 2 (18.2%), 3 | 1 (4.2%), 1 | 3 (8.6%), 4 |
| Tachypnea (not panting) | 0 | 3 (12.5%), 4 | 3 (8.6%), 4 |
| Albumin, high | 2 (18.2%), 2 | 0 | 2 (5.7%), 2 |
| Creatinine, high | 1 (9.1%), 1 | 1 (4.2%), 2 | 2 (5.7%), 3 |
| Decreased AST | 0 | 2 (8.3%), 2 | 2 (5.7%), 2 |
| Decreased bilirubin | 0 | 2 (8.3%), 2 | 2 (5.7%), 2 |
| Decreased PDW | 2 (18.2%), 3 | 0 | 2 (5.7%), 3 |
| Euthanasia | 2 (18.2%), 2 | 0 | 2 (5.7%), 2 |
| Hematuria | 0 | 2 (8.3%), 2 | 2 (5.7%), 2 |
| Lethargy | 0 | 2 (8.3%), 2 | 2 (5.7%), 2 |
| Lymphadenopathy | 1 (9.1%), 1 | 1 (4.2%), 1 | 2 (5.7%), 2 |
| Magnesium, high | 1 (9.1%), 1 | 1 (4.2%), 1 | 2 (5.7%), 2 |
| MCHC, low | 1 (9.1%), 1 | 1 (4.2%), 1 | 2 (5.7%), 2 |
| MPV, low | 2 (18.2%), 2 | 0 | 2 (5.7%), 2 |
| Muscle weakness, generalized or specific area | 0 | 2 (8.3%), 2 | 2 (5.7%), 2 |
| Neutropenia | 0 | 2 (8.3%), 2 | 2 (5.7%), 2 |
| Proteinuria | 0 | 2 (8.3%), 2 | 2 (5.7%), 2 |
| Sneezing | 0 | 2 (8.3%), 2 | 2 (5.7%), 2 |
| Tremors | 1 (9.1%), 1 | 1 (4.2%), 1 | 2 (5.7%), 2 |
| Triglycerides, high | 0 | 2 (8.3%), 3 | 2 (5.7%), 3 |
| Uveitis | 0 | 2 (8.3%), 2 | 2 (5.7%), 2 |
| A/G ratio increased | 0 | 1 (4.2%), 1 | 1 (2.9%), 1 |
| Abdominal distention | 0 | 1 (4.2%), 1 | 1 (2.9%), 1 |
| Aerophagia | 0 | 1 (4.2%), 6 | 1 (2.9%), 6 |
| Alopecia | 1 (9.1%), 1 | 0 | 1 (2.9%), 1 |
| Ascites | 0 | 1 (4.2%), 1 | 1 (2.9%), 1 |
| Detached retinas bilaterally with secondary  retinal degeneration | 0 | 1 (4.2%), 1 | 1 (2.9%), 1 |
| Bacteruria | 0 | 1 (4.2%), 1 | 1 (2.9%), 1 |
| Bilirubinemia altered liver function | 1 (9.1%), 1 | 0 | 1 (2.9%), 1 |
| Calcium, high | 0 | 1 (4.2%), 1 | 1 (2.9%), 1 |
| Chloride, high | 0 | 1 (4.2%), 1 | 1 (2.9%), 1 |
| CK, low | 0 | 1 (4.2%), 1 | 1 (2.9%), 1 |
| Conjunctivitis | 0 | 1 (4.2%), 1 | 1 (2.9%), 1 |
| Decreased amylase | 0 | 1 (4.2%), 1 | 1 (2.9%), 1 |
| Decreased plasma protein | 1 (9.1%), 1 | 0 | 1 (2.9%), 1 |
| Decreased sodium | 0 | 1 (4.2%), 1 | 1 (2.9%), 1 |
| Decreased sodium/potassium ratio | 0 | 1 (4.2%), 1 | 1 (2.9%), 1 |
| Depressed | 0 | 1 (4.2%), 1 | 1 (2.9%), 1 |
| Edema | 0 | 1 (4.2%), 1 | 1 (2.9%), 1 |
| Extremity (gait/ambulation) lameness | 1 (9.1%), 2 | 0 | 1 (2.9%), 2 |
| Flatulence | 0 | 1 (4.2%), 1 | 1 (2.9%), 1 |
| Fractured tooth | 0 | 1 (4.2%), 1 | 1 (2.9%), 1 |
| General performance | 0 | 1 (4.2%), 1 | 1 (2.9%), 1 |
| Heart murmur | 1 (9.1%), 1 | 0 | 1 (2.9%), 1 |
| Hematemesis | 0 | 1 (4.2%), 1 | 1 (2.9%), 1 |
| Increased basophils | 0 | 1 (4.2%), 1 | 1 (2.9%), 1 |
| Increased plasma protein | 0 | 1 (4.2%), 1 | 1 (2.9%), 1 |
| Increased PSL | 0 | 1 (4.2%), 1 | 1 (2.9%), 1 |
| Increased RBC in urine | 0 | 1 (4.2%), 1 | 1 (2.9%), 1 |
| Infection | 0 | 1 (4.2%), 1 | 1 (2.9%), 1 |
| Lameness | 0 | 1 (4.2%), 1 | 1 (2.9%), 1 |
| Limping | 1 (9.1%), 1 | 0 | 1 (2.9%), 1 |
| Listless | 0 | 1 (4.2%), 1 | 1 (2.9%), 1 |
| MCH, low | 1 (9.1%), 1 | 0 | 1 (2.9%), 1 |
| MPV, high | 0 | 1 (4.2%), 1 | 1 (2.9%), 1 |
| Nasal congestion | 0 | 1 (4.2%), 1 | 1 (2.9%), 1 |
| Nausea | 0 | 1 (4.2%), 1 | 1 (2.9%), 1 |
| Ocular discharge | 0 | 1 (4.2%), 1 | 1 (2.9%), 1 |
| Occult blood in urine | 0 | 1 (4.2%), 1 | 1 (2.9%), 1 |
| Panting, excessive | 0 | 1 (4.2%), 2 | 1 (2.9%), 2 |
| Phosphorus, high | 0 | 1 (4.2%), 1 | 1 (2.9%), 1 |
| Polydipsia | 0 | 1 (4.2%), 1 | 1 (2.9%), 1 |
| Polyuria | 0 | 1 (4.2%), 1 | 1 (2.9%), 1 |
| Potassium, high | 0 | 1 (4.2%), 1 | 1 (2.9%), 1 |
| Polydipsia | 0 | 1 (4.2%), 1 | 1 (2.9%), 1 |
| Prolonged prothrombin time | 1 (9.1%), 1 | 0 | 1 (2.9%), 1 |
| Restlessness | 0 | 1 (4.2%), 1 | 1 (2.9%), 1 |
| Retinal detachment | 0 | 1 (4.2%), 1 | 1 (2.9%), 1 |
| Seizure | 1 (9.1%), 1 | 0 | 1 (2.9%), 1 |
| Soft stool | 0 | 1 (4.2%), 1 | 1 (2.9%), 1 |
| Splenomegaly | 0 | 1 (4.2%), 1 | 1 (2.9%), 1 |
| Tachypnea | 0 | 1 (4.2%), 1 | 1 (2.9%), 1 |
| Urine pH, high | 0 | 1 (4.2%), 1 | 1 (2.9%), 1 |
| Urticaria (hives, welts, wheals) | 0 | 1 (4.2%), 1 | 1 (2.9%), 1 |
| Vocalizing | 1 (9.1%), 1 | 0 | 1 (2.9%), 1 |
